# Resonant multilevel optical switching with phase change material GST

**DOI:** 10.1515/nanoph-2022-0276

**Published:** 2022-06-29

**Authors:** Di Wu, Xing Yang, Ningning Wang, Liangjun Lu, Jianping Chen, Linjie Zhou, B. M. Azizur Rahman

**Affiliations:** Key Laboratory of Advanced Optical Communication Systems and Networks, Shanghai Key Lab of Navigation and Location Services, Department of Electronic Engineering, Shanghai Jiao Tong University, Shanghai 200240, China; Key Laboratory of Advanced Optical Communication Systems and Networks, Shanghai Key Lab of Navigation and Location Services, Shanghai 200240, China; School of Engineering & Mathematical Sciences, City University of London, London EC1V 0HB, UK

**Keywords:** integrated optics, optical memristive switch, phase change material, silicon photonics

## Abstract

We demonstrate a multilevel optical memristive switch based on a silicon Fabry–Perot resonator. The resonator is constructed by a pair of waveguide Bragg gratings at the ends of a multimode interferometer (MMI) covered with sub-micrometer-size Ge_2_Sb_2_Te_5_ (GST) thin film on top. The interaction between the optical field and GST is greatly enhanced due to the resonant effect. The GST phase transition is triggered by applying electrical pulses to the doped-silicon microheater. Light is transmitted when GST is amorphous while it is highly absorbed by the crystalline GST at the resonance wavelength, leading to a higher on-off extinction ratio (ER) compared to the non-resonant device. The resonant device achieves a maximum transmission contrast of 10.29 dB and a total of 38 distinct nonvolatile switching levels. Our work provides an effective solution to improving the multilevel switching performance of phase-change devices and paves the way for future nonvolatile silicon photonics devices.

## 1 Introduction

Silicon photonics combines the low-cost complementary metal-oxide-semiconductor (CMOS) compatible manufacturing technology with photonic technologies to deal with optical signal processing and transmission, which has become a research hotspot in both academia and industry [1]. Switching is a fundamental operation and is commonly implemented by refractive index (RI) modulation in photonic devices. Traditional silicon photonic devices use the free-carrier plasma dispersion (FCD) or the thermo-optic (TO) effects to realize RI modulation. However, the relatively weak RI tuning mechanism results in a long active waveguide length. Moreover, both the FCD and TO effects require a constant power supply to retain the desired RI change, resulting in considerable static power consumption. Therefore, it has become a bottleneck for further scaling down the active devices and reducing the power consumption.

Phase change materials (PCMs), such as Ge_2_Sb_2_Te_5_ (GST), are capable of reversible conversion between the amorphous (am) and the crystalline (cr) states and possess pronounced contrast in electrical and optical properties [2–4]. Introducing GST into silicon photonic devices using hybrid integration can provide a wide range of effective RI adjustment in a minimal volume, thus allowing for high-density integration and low power consumption [5–7]. Another advantage of GST lies in its non-volatility, which requires no power to maintain any particular state [8]. It has also been demonstrated that phase change can be triggered thermally [9, 10], optically [11–15], or electrically [16–18]. Owing to these merits of GST, it has been widely explored for various applications including optical switching [19–21], nonvolatile memory [22–24], and optical computing [25–27]. With a controlled volume ratio between the crystalline GST (cr-GST) and the amorphous GST (am-GST), multilevel optical switching with steady intermediate states is possible. The different switching levels can represent the storage bits in the photonic memories [22] or the synaptic weights in the optical neural networks [28]. The number of distinguishable switching levels is limited by the maximum transmission contrast between “ON” and “OFF” states, i.e., the switching extinction ratio (ER). Most of the reported multilevel switching devices have a low ER, and hence the functionality and the scalability are hard to be further improved. Although a higher ER can be obtained by increasing the volume of the GST cell, it degrades the operation speed and the repeatability of the phase change process [29]. In 2021, Wu et al. demonstrated a programmable phase-change metasurface on a silicon nitride waveguide with a high ER [30]. However, the phase transition is triggered optically. The active region and directional coupler for mode conversion are relatively long, which increases the device footprint.

In this work, we demonstrate an effective way to improve the ER by utilizing a Fabry–Perot (F-P) resonant cavity. Resonant structures can enhance light–matter interaction and increase optical absorption by the lossy medium [31]. By combining this feature with the large RI change and the partial crystallization ability of the GST, a multilevel electro-thermal switch with a compact volume and a high ER is implemented. Unlike the previously reported PCM switching devices based on microring resonators, there is no crucial requirement for our device to operate at the critical coupling to attain a high ER. The increased loss of crystalline GST is helpful to switch off light transmission since light inevitably goes through the GST absorption region. Benefiting from the multimode interferometer (MMI) structure, we integrated a silicon resistive microheater to electrically control the device state within a compact volume. The experiment reveals that the number of transmission levels can be increased considerably by the introduction of the F-P resonator compared to the non-resonant device.

## 2 Device structure and working principle

**Figure 1:**
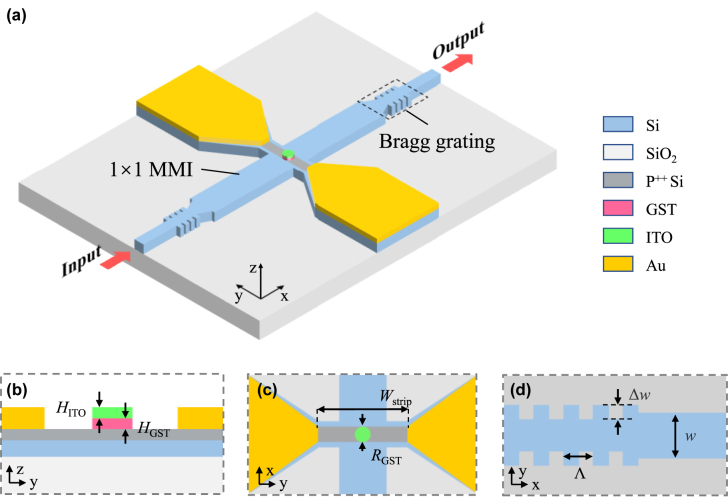
Schematic diagram of the proposed resonant optical memristive switch. (a) Three-dimensional perspective view of the optical memristive switch. (b) Cross-sectional view of the active region in the center of the MMI. (c) Top view of the device active region. (d) Top view of the Bragg grating mirror.

Figure 1a illustrates the schematic structure of the resonant optical memristive switch. It is composed of a 1 × 1 MMI inserted in an F-P cavity. The input/output is a single-mode silicon strip waveguide with a cross-sectional size of 220 nm (height) × 500 nm (width). The dimension of the MMI is 12.4 μm × 2 μm. Sidewall Bragg gratings form the front and rear mirrors of the F-P cavity. In the center of the MMI, a crossing strip with a 1 μm wide P^++^-doped region is placed perpendicularly. Gold (Au) electrodes are positioned on the two sides and the separation distance is *W*_strip_ = 3 μm. A thin film of GST with a disk shape is placed on top of the MMI at the crossing center. The GST thickness is *H*_GST_ = 30 nm and the disk radius is *R*_GST_ = 0.5 μm. To protect the GST film, another 30 nm-thick indium-tin-oxide (ITO) film covers the GST on top. The phase change is induced by resistive heating when an electrical pulse is applied. Figure 1b and c shows the cross-sectional view and top view of the active region, respectively. Figure 1d shows the structure of the Bragg grating reflector. The sidewall grating has a duty cycle of 50%. The Bragg resonance condition can be expressed as:

(1) $$\lambda =\frac{2\Lambda n_{\text{eff}}}{m}$$

where Λ is the grating period, *n*_eff_ is the effective refractive index of the grating waveguide, and *m* is the diffraction order (*m* = 1 in our design). The grating corrugation width is set to Δ*w* = 200 nm and the grating period is calculated to be Λ = 337 nm in order to set the Bragg resonance wavelength at *λ* = 1550 nm.

To analyze the output characteristics of the resonant switch, the GST-gated MMI is regarded as a waveguide with a variable loss (change with the GST phase state)*.* Then, the transmittance of the resonant switch can be expressed as [32]:

(2) $$T=\frac{I_{\text{t}}}{I_{0}}=\frac{{\left(1-R_{\text{g}}\right)}^{2}\alpha }{{\left(1-R_{\text{g}}\alpha \right)}^{2}+4R_{\text{g}}\alpha \sin^{2}\left(\phi +\phi_{\text{r}}\right)}$$

where $R_{g}={\left|r_{g}\right|}^{2}$ is the power reflectivity of the Bragg grating, *ϕ*_r_ is the reflection phase of the Bragg grating, *ϕ* and *α* are the one-way phase and the power loss factor of the F-P cavity. The reflection coefficient *r*_g_ is expressed as:

(3) $$r_{\text{g}}=\frac{-\kappa \sin h\left(\gamma L\right)}{i\gamma \cos h\left(\gamma L\right)+\Delta \beta \sin h\left(\gamma L\right)}$$

where *L* is the grating length,*κ* is the coupling coefficient, $\gamma =\sqrt{\kappa^{2}-\Delta \beta^{2}}$ , and $\Delta \beta =2\pi n_{\text{eff}}\left(\lambda^{-1}-\lambda_{0}^{-1}\right)$ , *λ*_0_ is the Bragg wavelength. The loss factors for am-GST and cr-GST are distinct: *α*_am_ is close to 1 and *α*_cr_ is close to 0. The operating wavelength is set to the resonant wavelength at the amorphous state. The ER, defined as the peak transmission contrast between the am-GST and cr-GST states, is then derived as:

(4) $$ER=\frac{T_{\text{am }-\;\max}}{T_{\text{cr }-\;\max}}=\frac{\alpha_{\text{am}}{\left(1-R_{\text{g}}\alpha_{\text{cr}}\right)}^{2}}{\alpha_{\text{cr}}{\left(1-R_{\text{g}}\alpha_{\text{am}}\right)}^{2}}$$

**Figure 2:**
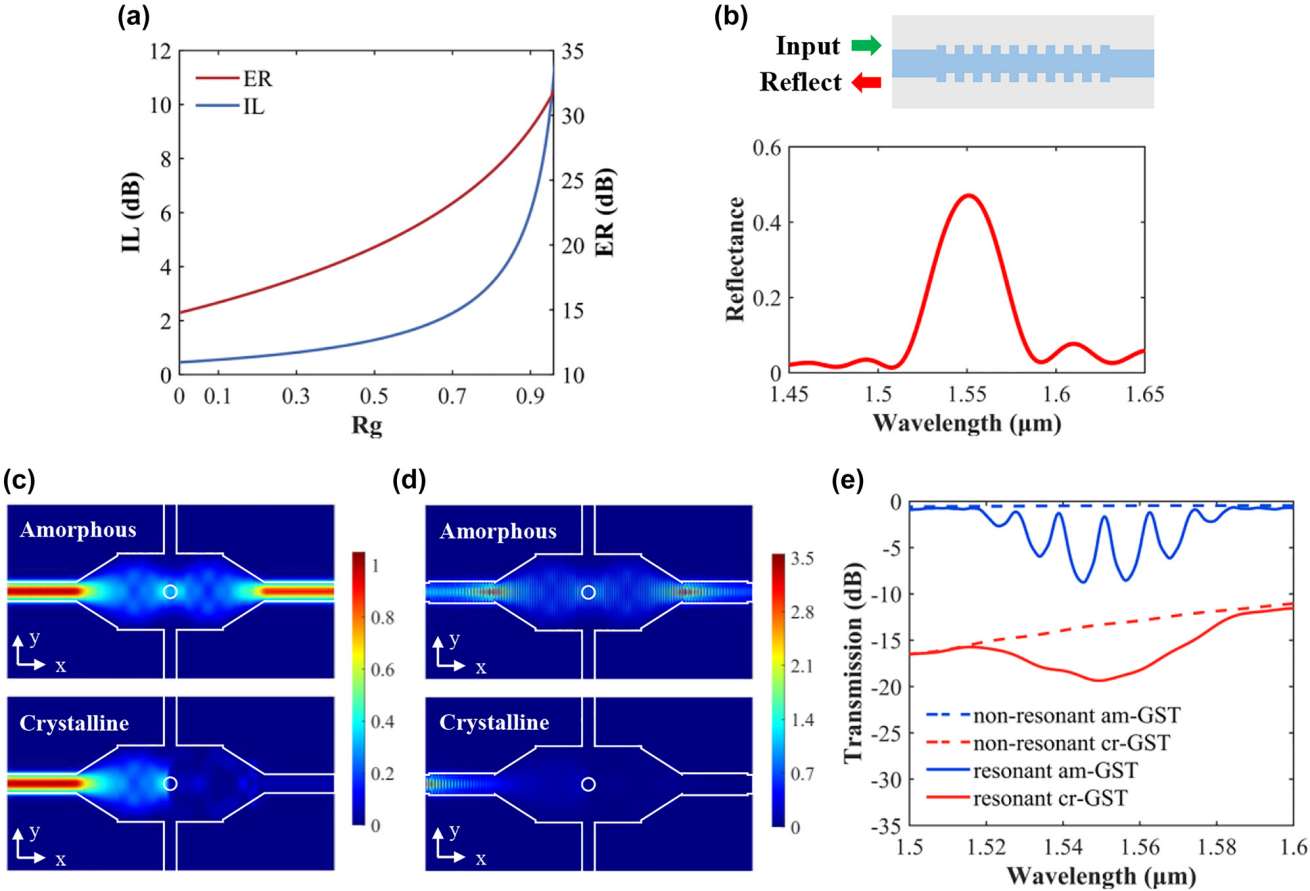
Theoretical calculation and simulation results of the device. (a) Calculated IL and ER as a function of Rg. (b) Simulated reflectance of the Bragg grating with the number of periods *N* = 20. (c, d) Simulated electric field intensity distributions in (c) the non-resonant switch and (d) the resonant switch. (e) Simulated transmission spectra of the non-resonant and resonant switches at am-GST and cr-GST states.

When there is no Bragg grating, the device becomes a non-resonant structure. In this case, *R*_g_ = 0 and ER = *α*_am_/*α*_cr_, which indicates the transmission contrast is dictated by the MMI loss variation upon phase change. When *R*_g_ increases to approach 1, the ER increases monotonously. It hence suggests that the introduction of the resonant cavity increases the transmission contrast. It should be noted that the insertion loss (IL) at the amorphous state is also increased. Figure 2a shows the calculated ER and IL as a function of the Bragg grating reflectivity. In the simulation, we assume *α*_am_ = 0.9 and *α*_cr_ = 0.03, consistent with the practical device. The grating reflectance in our device is chosen to be *R*_g_ = 0.5 to make a compromise between ER and IL.

We used the three-dimensional finite-difference time-domain (3D-FDTD) method to simulate and optimize the device design. By changing the number of grating periods, we can adjust the grating reflectance and hence the output transmission. The number of grating periods is calculated to be *N* = 20 to get *R*_g_ = 0.5 at the Bragg resonance wavelength. Figure 2b shows the calculated reflectance for the grating. The full width at the half maximum (FWHM) exceeds 40 nm. Figure 2c and d shows the electric field intensity distributions in the non-resonant and resonant switches, respectively. When GST is in the amorphous state with its complex refractive index being 3.98 + 0.0244*i*, the insertion loss of the device is low and the switch state is “ON”. When GST changes to the crystalline state with its complex refractive index increased to 6.49 + 1.054*i*, it possesses strong absorption and turns the switch state to “OFF”. The complex refractive indices were adopted from the measurement by spectroscopic ellipsometry. The F-P cavity generates a noticeable resonance in the MMI, which enhances light absorption at the MMI center in the crystalline state. As a result, the optical transmission contrast between two phase states at the Bragg resonance wavelength is largely enhanced.

Figure 2e shows the simulated transmission spectra of the non-resonant and resonant switches at the “ON” and “OFF” states. At the 1550 nm wavelength, the “ON” state loss of the non-resonant switch is 0.48 dB and the “OFF” state loss is 15.41 dB. The ER is 12.82 dB. In contrast, prominent resonance peaks can be observed for the resonant switch at the “ON” state. One of the F-P resonances occurs at 1550 nm. Light is more strongly absorbed by cr-GST in the cavity, leading to a reduced transmission level in the Bragg reflection band. At the 1550 nm wavelength, the “ON” state loss is 1.65 dB and the “OFF” state loss is 19.31 dB. The ER is 17.65 dB, increased by 4.8 dB compared to the non-resonant device.

## 3 Device fabrication

**Figure 3:**
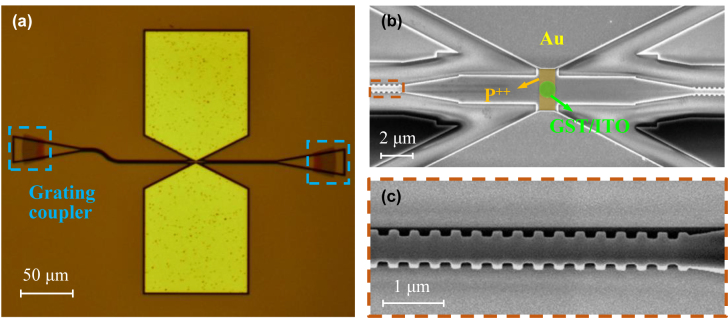
Fabricated devices. (a) Microscope image of the fabricated resonant memristive switch. (b, c) SEM images of (b) the MMI structure and (c) the Bragg grating.

The memristive switches were fabricated on a silicon-on-insulator (SOI) substrate with a 220 nm-thick top-silicon layer and a 2 µm-thick buried-oxide layer. The silicon waveguide patterns were first defined in the CSAR 6200.09 positive resist using a Vistec EBPG5200+ electron beam lithography (EBL) system. The patterns were transferred to the silicon layer by inductively coupled plasma (ICP) dry etching. Next, a 2 μm-thick positive-tone PMMA electron-beam resist was spin-coated and patterned to open the window for the following boron ion implantation. The ion implantation dose is 3 × 10^15^ ions/cm^2^. The chip was then annealed at 1100 °C for 15 s to activate the dopants after ion implantation. After that, a 1 μm-thick PMMA was spin-coated, and a third-time EBL was performed to define the GST deposition window. A 30 nm-thick GST film and a 30 nm-thick ITO protective film were deposited by magnetron sputtering. The sputtering conditions are 15 sccm Ar flow rate and 30 W RF power for GST deposition and 25 sccm Ar flow rate and 200 W DC power for ITO deposition. The GST/ITO films were patterned by the lift-off process. The metal electrodes were defined using a fourth-time EBL. In order to form a good ohmic contact with the doped silicon, the chip was immersed in a buffered oxide etchant (BOE, 10:1 HF (49%) and NH_4_F (10%)) for 15 s at ambient temperature to remove the thin native oxide before metal deposition. The metal layers consisting of 20 nm-thick Cr and 100 nm-thick Au were deposited using electron-beam evaporation followed by a lift-off process. Figure 3a shows the microscope image of the fabricated device. Figure 3b and c shows the scanning electron microscope (SEM) images of the MMI structure and the Bragg grating, respectively.

## 4 Measurement

We used a tunable laser source (Agilent 81600B) and an optical power monitor (Agilent 81636B) to measure the transmission spectrum. Continuous-wave (CW) from the laser source was adjusted to the quasi-transverse-electric (TE) mode through a polarization controller and then vertically coupled into the device through the grating coupler at an angle of about 10°. The coupling loss is ∼4–4.5 dB/facet at the 1550 nm wavelength. An arbitrary waveform generator (AWG, Agilent 81150A) was used to generate the electrical pulses, which were applied to the Au electrodes via a pair of metal probes.

**Figure 4:**
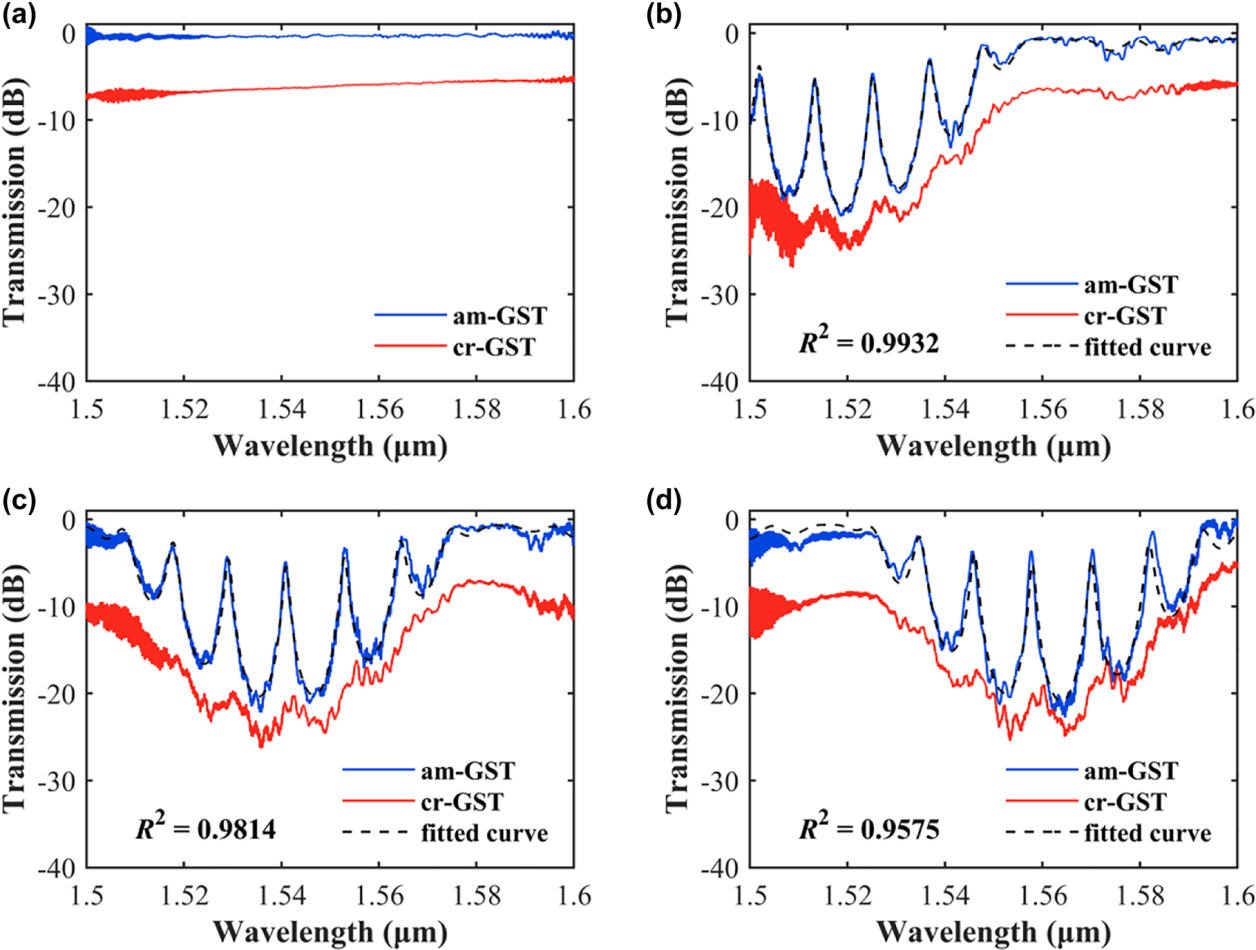
Measured transmission spectra of (a) the non-resonant switch and (b–d) the resonant switches with grating periods of (b) 337 nm, (c) 347 nm, and (d) 357 nm in am-GST and cr-GST states. The black dashed lines are the fitted curves of the spectra when the resonant devices are in the am-GST state.

Figure 4 shows the measured transmission spectra of three resonant switches with grating periods of 337 nm, 347 nm, and 357 nm, together with the non-resonant control device. The spectra were normalized to a reference straight waveguide. Due to the fabrication errors, the Bragg resonant wavelengths were blue-shifted compared to the simulation. The operation wavelengths of the three devices can be chosen as 1525.20 nm, 1540.89 nm, and 1557.69 nm to get the maximum transmission contrast. The as-fabricated devices are initially at the “ON” state, where GST is amorphous. In order to obtain the “OFF” state, the devices were heated on a hot plate at 180 °C temperature for 3 min to convert the initial am-GST to cr-GST. The “ON” and “OFF” state loss of the non-resonant switch at the 1550 nm wavelength is 0.37 dB and 6.13 dB, respectively. Therefore, the ER is calculated to be 5.76 dB. We select the resonant switch with a grating period of 357 nm for comparison. The “ON” state loss (IL) is 3.65 dB and the “OFF” state loss is 20.56 dB, resulting in an ER of 16.91 dB. It should be noted that the resonant devices have the same design as the non-resonant device expect the additional Bragg gratings. The above measurements clearly indicate the effectiveness of the ER enhancement by the F-P resonances.

Based on the theoretical model of Eq. (2), we can perform curve fitting of the measured spectra to extract the key parameters. The fitting matches well with the measured ones, as shown by the dashed black cures in Figure 4. The obtained coupling coefficient of the Bragg gratings is about 2000 cm^−1^ and the peak reflectance is 0.83, which is larger than our design. This deviation also causes the increased IL of our fabricated device, as predicted by Figure 2a.

The non-resonant MMI switch with a doped silicon microheater has been proved to have good repeatability and high performance in multilevel memory and logic operation [29]. We characterized the multilevel switching performance of the resonant switch following the same method. The response time of the amorphization and crystallization processes is around 100 ns. The initial state of the device is set to amorphous by using a pre-amorphization electrical pulse with a width of 20 ns and an amplitude of 9 V. The device output transmission is high (level 0) at the beginning.

**Figure 5:**
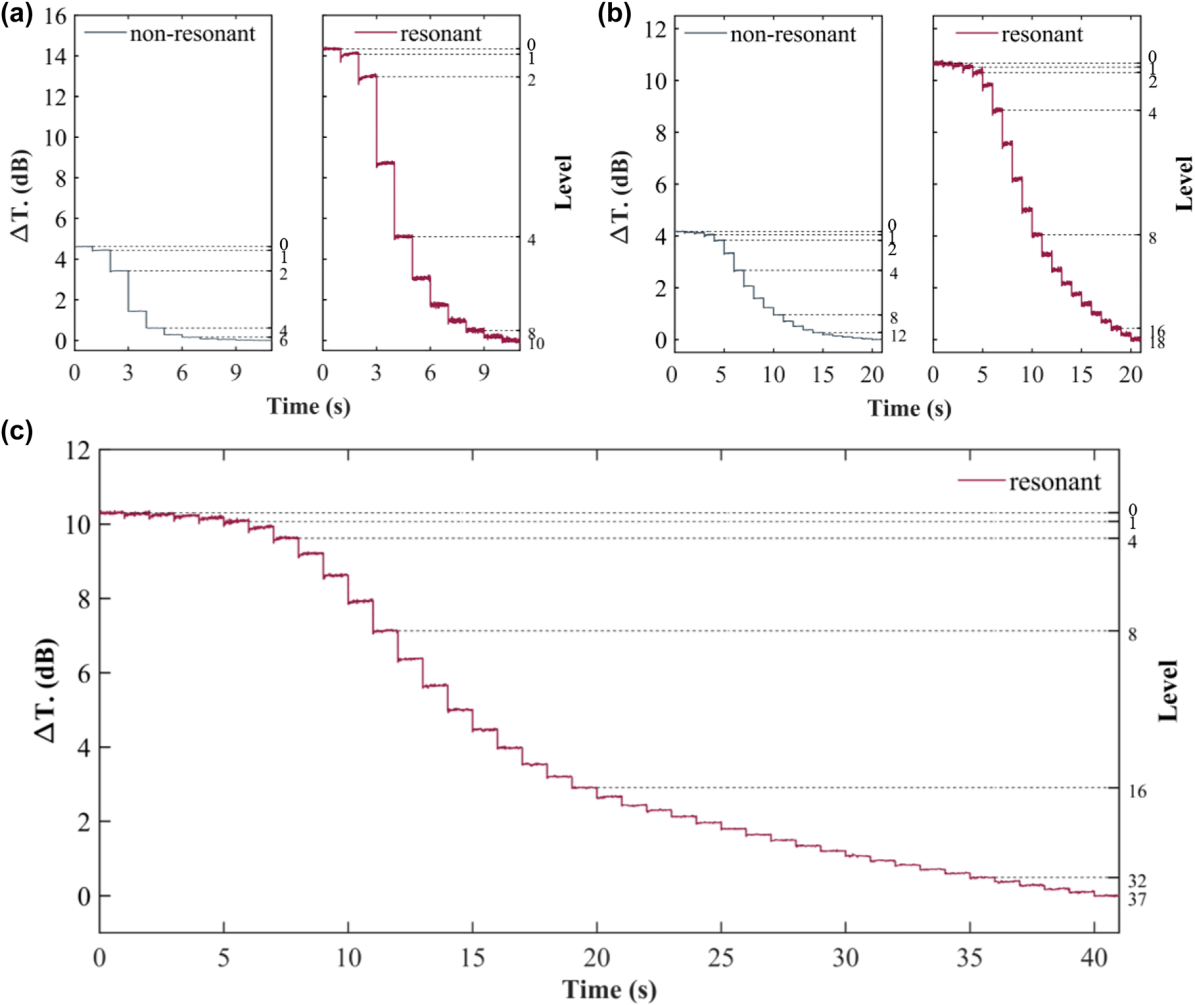
Multilevel switching performance. (a, b) Multilevel switching of the non-resonant switch and the resonant switch using single-step crystallization pulses with the pulse energy of (a) 4.08 nJ, and (b) 3.85 nJ. (c) Multilevel switching of the resonant switch using single-step crystallization pulses with the pulse energy of 3.40 nJ.

Starting from level 0, we performed the stepwise crystallization using consecutive single-step electrical pulses. Each pulse is temporally separated by 1 s. The output power from the device was monitored by a photodetector. Figure 5a shows the normalized transmission change under a sequence of crystallization pulses with a width of 120 ns and an amplitude of 3.5 V. Since the measured resistance of the devices is approximately 360 Ω, the applied pulse energy is ∼4.08 nJ. The resonant switch with a 357 nm grating period was chosen for this experiment. The other two devices have similar performances. The normalized transmission change is defined as Δ*T* = 10 log_10_(*T*/*T*_0_), where *T* is the output power for a particular transmission level and *T*_0_ is the output power for the lowest transmission (GST nearly fully crystallized) as the baseline. The resolvable transmission levels are limited by the noise level of the photodetector. We used 0.1 dB as the minimum distinguishable output difference for decision. We can see that the transmission difference between the two adjacent output levels first increases and then decreases. For the non-resonant switch, there are 7 distinguishable switching levels. For the resonant switch, the number of switching levels increases to 11 in the crystallization process. Due to the higher ER of the resonant switch, each phase change step produces a more pronounced change in the transmission than the non-resonant switch. The total “ON-OFF” transmission contrast of the resonant switch is 14.39 dB, which is much larger than the 4.64 dB contrast of the non-resonant switch.

Next, we reduced the amplitude of the electrical pulses to 3.4 V and re-performed the multilevel optical switching. The pulse energy was reduced to 3.85 nJ. Figure 5b shows the transmission change with time. The first three crystallization pulses do not induce significant change to the output transmission, probably due to the low nucleation rate caused by the decreased temperature in the GST layer [33, 34]. Nevertheless, the total number of distinguishable switching levels increases to 13 and 19 for the non-resonant and resonant switches, respectively. The “ON-OFF” transmission contrast is 4.16 dB and 10.62 dB for the non-resonant and resonant switches, respectively, which is lower than the previous higher-energy operation.

There is still room to insert more levels as can be seen in Figure 5b. Figure 5c shows the stepwise transmission of the resonant switch when the electrical pulse energy is reduced to 3.40 nJ by setting the pulse width to 100 ns and the amplitude to 3.5 V. The first four steps are hard to distinguish due to the reduction of the electrical pulse energy. After that, the transmission level drop is significant. There is a total of 38 observable switching levels with a maximum transmission contrast of 10.29 dB.

## 5 Discussion

Benefitting from the enhanced ER of the resonant switch, we can obtain more transmission levels generated by consecutive crystallization pulses, which outperforms the non-resonant switch. Due to the phase change properties of GST material, when the applied pulse energy is low, the nucleation rate of crystallization is slow, and multiple pulses are required to switch the device from level 0 to level 1. Since the low pulse energy limits the maximum temperature of the GST thin film in the crystallization process, increasing the pulse number cannot achieve fully crystalline GST. By programming the electrical pulses, such as progressively increasing the phase change pulse energy, it is possible to increase the ratio of the cr-GST and generate more evenly distributed intermediate levels, further increasing the number of output levels. Employing a low-noise photodetector can also improve the resolved transmission levels.

Although we have performed the switching in a stepwise manner, the transition between any level can be easily achieved. When switching from a lower-level *x* (*x* ≥ 0) to an arbitrary higher-level *y* (*y* > *x*), we can use a set of *y*–*x* crystallization pulses. When switching from a higher-level *y* to an arbitrary lower-level *x*, we can first use an amorphization pulse with higher energy to reset the device state to level 0, then switch to level *x* with *x* crystallization pulses. The transmission state of the switch can likewise be changed by applying crystallization pulses with different energies. It is also possible to use one double-step pulse instead of multiple stepwise pulses for switching between arbitrary levels [25]. The double-step pulse is composed of a high-energy step to erase the previous state followed by a low-energy step to crystallize and determine the final transmission level. By combining amorphization and crystallization processes, transmission levels could be switched by a single programmed pulse, which further improves the switching speed of the device.

Table 1 compares this work with other reported multilevel switching devices based on PCMs. To facilitate the comparison, we also list the relative transmission change in percentage as adopted by some work in the literature, which is expressed as Δ*T*_linear_ = (*T* − *T*_0_)/*T*_0_. Our device achieves the maximum transmission contrast and the highest number of switching levels among the electrically triggered multilevel switching devices. Although optically induced phase change could support all-optical operation and high transmission contrast, it is more suitable for individual devices or small integration since it has to use separate pump light to trigger the phase change of GST. The routing complexity of the pump light increases considerably with the number of devices to be controlled. This makes it challenging to be used in large-scale photonic integrated circuits [20, 37]. In comparison, the electrically induced phase transition has more convenience to be applied in large-scale integration. The micro-heaters can be integrated anywhere phase change is required without intervening in the optical paths. It can be potentially applied to large-scale optical switches, reconfigurable optical processors, and neuromorphic optical computers, etc., where the elementary optical components need to be driven electrically.

**Table 1:** Comparison of reported multilevel switching devices.

Ref.	Phase change method	Max trans. contrast (Δ*T*_linear_)	# Of levels
[24]	Optically	0.83 dB (21%)	8
[35]	Optically	0.79 dB (20%)	11
[23]	Optically	1.37 dB (37%)	34
[30]	Optically	16.5 dB (4369%)	64
[36]	Electrically & optically	0.03 dB (0.7%, elec.)	–
		0.19 dB (4.5%, opt.)	
[29]	Electrically	6.02 dB (300%)	5
[17]	Electrically	2.23 dB (67%)	20
This work	Electrically	10.29 dB (969%)	38

## 6 Conclusions

We have demonstrated a resonance-enhanced multilevel optical memristive switch based on the phase change material GST. F-P resonances are generated due to the presence of Bragg gratings at the input and output ends of the MMI. Electrical pulses are applied to the silicon resistive microheater to trigger the phase change of the GST cell on top of the MMI. We use consecutive single pulses to perform stepwise crystallization to study the multilevel switching capability of resonant and non-resonant devices. It reveals that the resonant device has a much larger transmission change and possesses more distinguishable intermediate states. We achieve a maximum transmission contrast of 10.29 dB with 38 output levels. Benefiting from the nonvolatile property of GST material, our device can be used in multilevel optical memory, in-memory computing [26], and neuromorphic computing [38] with compact size and low power consumption. This work represents a significant step toward low-power, high-speed, and highly integrated on-chip reconfigurable devices on the silicon photonics platform.
